# Human iPSC-derived motor neurons as a platform for elucidating TDP-43-related amyotrophic lateral sclerosis pathogenesis: a mini review

**DOI:** 10.3389/fnmol.2026.1864964

**Published:** 2026-05-22

**Authors:** Satoshi Yokoi, Yohei Iguchi, Masahisa Katsuno

**Affiliations:** 1 Department of Pathophysiological Laboratory Sciences, Nagoya University Graduate School of Medicine, Nagoya, Japan; 2 Department of Neurology, Nagoya University Graduate School of Medicine, Nagoya, Japan

**Keywords:** amyotrophic lateral sclerosis, co-culture, iPSC-derived motor neuron, RNA-binding protein, TDP-43

## Abstract

TAR DNA-binding protein 43 (TDP-43) is a major pathogenic RNA-binding protein associated with amyotrophic lateral sclerosis (ALS). Heterozygous mutations in TDP-43 cause familial ALS, known as ALS10. TDP-43 is predominantly localized in the nucleus under physiological conditions. Not only ALS patients with *TARDBP* mutations but also the majority of sporadic ALS patients exhibit TDP-43 pathology, which is defined by nuclear clearance and cytoplasmic aggregation. The inclusion of cryptic exons in genes such as STMN2 and UNC13A has emerged as a hallmark of TDP-43 loss of function, as demonstrated in TDP-43 knockdown models and postmortem analyses. However, it is not yet clear how TDP-43 levels and location change from healthy to pathological conditions in ALS. Motor neurons derived from induced pluripotent stem cells (iPSCs) have been widely used in ALS research and provide a promising platform to investigate early-stage disease mechanisms. However, challenges remain in generating models that faithfully recapitulate ALS pathogenesis. In this review, we summarize recent advances in TDP-43-related iPSC-derived motor neuron models and discuss future perspectives for elucidating ALS pathogenesis. We propose that longitudinal analyses of TDP-43 dynamics and co-culture systems will be essential to better model early ALS pathogenesis.

## 1 Introduction

Amyotrophic lateral sclerosis (ALS) is a progressive neurodegenerative disorder characterized by the degeneration of both upper and lower motor neurons (Brown and Al-Chalabi, 2017). Approximately 10% of ALS cases are familial, with more than 30 causative genes identified, while the remaining ~90% are sporadic. TAR DNA-binding protein 43 (TDP-43), encoded by the *TARDBP* gene, is a major RNA-binding protein in RNA processing (Tollervey et al., 2011; Lagier-Tourenne et al., 2012; Alami et al., 2014; Piol et al., 2023; Arnold et al., 2025). Under physiological conditions, TDP-43 is predominantly localized in the nucleus; however, in ALS, it is mislocalized to the cytoplasm, where it accumulates pathologically with phosphorylation (Arai et al., 2006; Neumann et al., 2006). Phosphorylated TDP-43, particularly at Ser409/410, is one of the most reproducible and widely used pathological markers of ALS (Hasegawa et al., 2008). Although other post-translational modifications such as ubiquitination (Neumann et al., 2006), SUMOylation (Maurel et al., 2020), acetylation (Cohen et al., 2015), and citrullination (Arseni et al., 2023) have been reported, multiple experimental studies have linked TDP-43 phosphorylation to cytoplasmic aggregation, cellular toxicity, and neurodegeneration (Liachko et al., 2014; Nonaka et al., 2016; Ko et al., 2024). Abnormal TDP-43 mislocalization and aggregation are primarily observed in the neuronal cytoplasm, dystrophic neurites, and glial cytoplasm (Arai et al., 2006; Neumann et al., 2006). In experimental models, mislocalized or aggregated TDP-43 has additionally been associated with stress granules (Liu-Yesucevitz et al., 2010; McDonald et al., 2011), mitochondria (Wang et al., 2016), and the endoplasmic reticulum (Kon et al., 2022; Wu et al., 2024), suggesting the involvement of multiple subcellular compartments in TDP-43-mediated neurodegeneration. This TDP-43 pathology is observed in approximately 97% of ALS cases, including sporadic forms (Ling et al., 2013), highlighting its central role in ALS pathogenesis. Consistently, heterozygous mutations in *TARDBP* cause familial ALS, designated ALS10 (Kabashi et al., 2008; Sreedharan et al., 2008), further underscoring the importance of TDP-43 in disease development. Notably, patients with *TARDBP* mutations exhibit TDP-43 pathology similar to that observed in sporadic ALS, suggesting that investigating disease mechanisms driven by *TARDBP* mutations may provide insights into the pathogenesis of the vast majority of ALS cases, including sporadic forms. Interestingly, TDP-43 pathology is also observed in approximately half of frontotemporal dementia (FTD) cases, a major cause of young-onset dementia characterized by progressive deterioration in behavior or language (Neumann et al., 2006; Ling et al., 2013). *TARDBP* mutations can cause FTD, supporting the concept that ALS and FTD represent overlapping TDP-43 proteinopathies.

Recent advances in ALS research have identified cryptic exon inclusion in genes such as Stathmin-2 (STMN2) and the UNC-13 homologs UNC13A as a hallmark of TDP-43 loss of function associated with nuclear depletion of TDP-43 (Klim et al., 2019; Melamed et al., 2019; Brown et al., 2022; Ma et al., 2022). STMN2 regulates normal axonal outgrowth and regeneration. Cryptic exon inclusion of STMN2 induces premature polyadenylation and generates a truncated STMN2 transcript, resulting in reduced full-length STMN2 expression. Loss of STMN2 has been observed in postmortem spinal cord tissue of ALS patients (Klim et al., 2019). In addition, suppression of STMN2 cryptic exon inclusion rescued STMN2 expression and ameliorated phenotypes induced by TDP-43 depletion, including deficits in neurite outgrowth and axon regeneration (Baughn et al., 2023). These data indicate that STMN2 may play an important role in ALS pathogenesis. UNC13A regulates synaptic vesicle priming and neurotransmitter release (Dittman, 2019). Cryptic exon inclusion in UNC13A induces nonsense-mediated decay, resulting in reduced UNC13A expression. Moreover, ALS/FTD-associated *UNC13A* risk variants increase cryptic exon inclusion, highlighting a strong mechanistic link between TDP-43 loss of function and ALS/FTD pathogenesis (Brown et al., 2022; Ma et al., 2022). Importantly, the presence of cryptic exon inclusion in postmortem samples strongly supports the relevance of TDP-43 loss of function in ALS pathogenesis, particularly in advanced disease stages. Based on these findings, clinical trials of splice-switching antisense oligonucleotides targeting STMN2 are currently underway in ALS patients (Genge et al., 2023). However, it remains unclear how TDP-43 levels and subcellular localization change during the transition from physiological to pathological states in ALS.

Despite the development of transgenic and knock-in mouse models, recapitulating progressive TDP-43 pathology and early disease mechanisms remains challenging. In addition, RNA-binding proteins often exhibit species-specific RNA-binding profiles (Yokoi et al., 2022; Harris et al., 2024), limiting the ability of rodent models to fully capture human-specific RNA regulatory mechanisms. To overcome these limitations, motor neuron models derived from human induced pluripotent stem cells (iPSCs) provide a promising platform for investigating early-stage disease mechanisms in a human context. Drug screening studies using iPSC-derived motor neurons (iPSMNs) from ALS patients have identified candidate compounds (Egawa et al., 2012; Fujimori et al., 2018; Bye et al., 2026), which several studies have progressed to clinical trials (Imamura et al., 2019; Morimoto et al., 2023). In addition, large-scale multi-omics resources such as Answer ALS have accelerated the identification of disease-associated molecular signatures in patient-derived iPSMNs (Baxi et al., 2022). Nevertheless, significant challenges remain in developing models that accurately recapitulate ALS pathogenesis, as culture conditions can substantially influence disease phenotypes. In this review, we summarize recent advances in TDP-43-related iPSC-derived motor neuron models and discuss future perspectives for elucidating ALS pathogenesis.

## 2 The overview of phenotypes in iPSC-derived motor neuron models with *TARDBP* mutations

To better understand TDP-43-related pathogenic mechanisms, we reviewed studies using iPSMN models with *TARDBP* mutations, including N290S, G287S, G298S, A315T, M337V, Q343R, G348C, A382T, I383T, and I383V (Bilican et al., 2012; Egawa et al., 2012; Serio et al., 2013; Devlin et al., 2015; Fujimori et al., 2018; Klim et al., 2019; Dafinca et al., 2020; Fazal et al., 2021; Smith et al., 2021; Harley et al., 2023; Held et al., 2023; Onda-Ohto et al., 2023; Lépine et al., 2024; Chikuchi et al., 2025). These mutations are located in the C-terminal domain, also referred to as the glycine-rich or prion-like domain, which has been implicated in phase separation and TDP-43 aggregation (Tziortzouda et al., 2021). Most ALS-associated TARDBP mutations are located in this domain (Versluys et al., 2022). The mutations included in this review represent a subset of TARDBP mutations reported in ALS patients.

Changes in TDP-43 protein expression were inconsistent across studies. Several reports demonstrated increased total or insoluble TDP-43 expression (Bilican et al., 2012; Egawa et al., 2012; Fazal et al., 2021; Chikuchi et al., 2025). Likewise, TDP-43 localization varied among studies, although increased cytoplasmic TDP-43 was frequently observed (Egawa et al., 2012; Fujimori et al., 2018; Fazal et al., 2021; Smith et al., 2021; Held et al., 2023). We previously demonstrated increased nuclear TDP-43 (Chikuchi et al., 2025). Cytoplasmic aggregation of phosphorylated TDP-43 is a pathological hallmark of ALS; however, although phosphorylated TDP-43 was detected in iPSMNs, robust cytoplasmic aggregation resembling end-stage ALS pathology has not been consistently reproduced.

The neuronal phenotypes observed in *TARDBP* mutant iPSMNs also showed substantial variability. Several studies reported neuronal hyperexcitability (Devlin et al., 2015; Harley et al., 2023), whereas others observed hypoexcitability (Dafinca et al., 2020; Smith et al., 2021; Lépine et al., 2024; Chikuchi et al., 2025). These discrepancies were not consistently associated with changes in TDP-43 expression or localization, suggesting that the difference in *TARDBP* mutations or different culture conditions may influence neuronal phenotypes. Synaptic morphology was examined in two reports. *TARDBP* mutations were associated with increases in the number or size of synaptic puncta, indicating a potential role for *TARDBP* in synaptic function and plasticity (Lépine et al., 2024; Chikuchi et al., 2025). In addition, reduced STMN2 expression or STMN2 cryptic exon inclusion have been documented in only two studies (Klim et al., 2019; Smith et al., 2021), and these alterations were not necessarily accompanied by overt nuclear loss of TDP-43. Compared with STMN2, evidence for UNC13A dysregulation in *TARDBP*-mutant iPSMNs remains limited.

Importantly, the phenotypes exhibited variation in the healthy iPSC lines used for the insertion of *TARDBP* mutations. Smith et al. utilized two different healthy iPSC lines for *TARDBP* mutation knock-in and found that increased cytoplasmic TDP-43 and STMN2 cryptic exon inclusion were observed only in one line, whereas neuronal firing changes showed opposite trends between the two lines (Smith et al., 2021). These findings indicate that the genetic background of the healthy iPSC lines should be carefully considered when generating mutation-inserted models.

Only two studies incorporated co-culture with astrocytes. These studies demonstrated that co-culture with astrocytes enhanced synaptic maturation or neuronal activity (Serio et al., 2013; Chikuchi et al., 2025). Importantly, Serio et al. demonstrated that the vulnerability of *TARDBP* mutant iPSMNs observed under monoculture conditions was rescued by co-culture with either wild-type (WT) or *TARDBP* mutant astrocytes. This study suggests that the enhancement of neuronal function or cell viability through co-culture with astrocytes may mask subtle mutation-associated phenotypes.

Collectively, previous studies indicate that *TARDBP* mutant iPSMN models can reproducibly capture early functional abnormalities rather than overt end-stage TDP-43 pathology. At the same time, the marked variability in TDP-43 expression, localization, and neuronal phenotypes across models underscores the need to improve iPSMN models to elucidate the disease course of ALS patients.

## 3 Discussion

### 3.1 Early TDP-43 dynamics as a key pathogenic event

TDP-43 loss from the nucleus, neuronal cytoplasmic inclusions, skein-like inclusions and glial inclusions are the hallmarks of TDP-43 pathology (Arai et al., 2006; Neumann et al., 2006; Neumann, 2009). These features were common in familial ALS with *TARDBP* mutations and sporadic ALS. However, temporal changes in TDP-43 expression, especially early-stage alterations in TDP-43 expression, have yet to be fully elucidated. *TARDBP* mutations were reported to enhance protein stability (Ling et al., 2010; Watanabe et al., 2013), indicating that increased TDP-43 expression may appear in the disease course. Indeed, we previously reported enhanced TDP-43 expression in the nucleus associated with altered neuronal activity in the iPSMN model with TDP-43 I383V mutation (Chikuchi et al., 2025). In TDP-43 knock-in mice, increased nuclear TDP-43 expression was also observed (White et al., 2018). Our previous findings further support the concept that enhanced nuclear TDP-43 expression may represent one of the early pathological changes in TDP-43 pathology. As mentioned above, several studies of *TARDBP* mutant iPSMN models showed increased TDP-43 expression. However, few reports have demonstrated where TDP-43 was indeed increased in subcellular compartments. As iPSMN models have the advantage of the analysis of early phenotypes, precise evaluations of TDP-43 expression and its localization are important to understand the initiation of TDP-43 pathology.

Longitudinal analysis of TDP-43 expression and associated neuronal phenotypes may provide valuable insights into iPSMN models. Harley et al. demonstrated that TDP-43 mutant iPSMNs exhibited elongated axonal initiation sites (AISs) and increased intrinsic hyperexcitability following a two-week culture period (Harley et al., 2023). In this period, TDP-43 expression remained unchanged. Interestingly, these phenotypes underwent a reversal over the course of a six-week culture period, accompanied by a decline in TDP-43 expression in the nucleus. Furthermore, shortened AISs were observed in postmortem ALS spinal cords, suggesting that nuclear TDP-43 loss may be a hallmark of advanced stages of the disease. This research underscores the significance of examining temporal shifts in TDP-43 expression and neuronal phenotypes. Such longitudinal analysis is a major strength of iPSMN models and is important for understanding early ALS pathogenesis.

### 3.2 The importance of co-culture systems

Co-culture in iPSMN models has the potential to promote physiological neuronal conditions and to elucidate pathogenic mechanisms more precisely. Astrocytes play important roles in structural support, functional integrity, and homeostasis in the brain. Astrocyte–neuron interactions are critical for the regulation of synapses and neural circuits (Chung et al., 2015; Ngoc et al., 2025). Indeed, several studies utilized co-culture of iPSC-derived astrocytes (Madill et al., 2017; Birger et al., 2019; Zhao et al., 2020) or rodent astrocytes (Wainger et al., 2014; Kim et al., 2020) for analyzing ALS pathogenesis. We also demonstrated that co-culture of iPSMNs with rat astrocytes promoted neuronal activity compared with monoculture (Chikuchi et al., 2025), and TDP-43 expression was increased in TDP-43 mutant iPSMNs as mentioned above. Further discussion is necessary to determine which astrocytes, whether primary rodent astrocytes or human iPSC-derived astrocytes, are suitable for iPSC-derived neuron culture (Lischka et al., 2018; Hedegaard et al., 2020; Lendemeijer et al., 2024). However, the promotion of physiological neuronal conditions through astrocyte co-culture may better reveal disease-relevant phenotypes in ALS iPSMN models. Microglia are implicated in both inflammatory responses to extracellular TDP-43 and the clearance of pathological TDP-43 (Spiller et al., 2018; Swanson et al., 2025). Although co-culture systems combining iPSC-derived motor neurons and iPSC-derived microglia have been established in other ALS-associated mutation models (Vahsen et al., 2023), studies using *TARDBP*-mutant iPSC-derived motor neurons remain limited.

The neuromuscular junction is also an important component affected in ALS. Co-culture with iPSC-derived skeletal muscle cells has been used to analyze the dysfunction of neuromuscular junctions (Fralish et al., 2021; Badu-Mensah et al., 2022). Moreover, 3D organoids have emerged to analyze the function of motor neurons and neuromuscular junctions. Osaki et al. developed a microfluidic device that connects iPSMN spheroids and iPSC-derived three-dimensional skeletal muscle bundles to form a motor unit that recapitulates functional neuromuscular junctions (Osaki et al., 2018). Compared to a non-ALS motor unit, the ALS motor unit with the TDP-43 G298S mutation demonstrated fewer muscle contractions, increased motor neuron degeneration, and increased apoptosis in skeletal muscle cells. Pereira et al. (2021) generated sensorimotor organoids containing physiologically functional neuromuscular junctions. Muscle contraction was reduced in ALS organoids, and the number of innervated NMJs was decreased in TDP-43 G298S mutant organoids.

Although further methodological improvements are needed to analyze ALS pathology, these advances indicate that co-culture methods have the potential for recapitulating physiological motor neuron conditions and neuromuscular junctions.

In summary, human iPSC-derived motor neurons have emerged as a valuable platform for investigating early TDP-43-related pathogenic mechanisms in ALS. Although further improvements in longitudinal analyses and co-culture systems are needed to more faithfully recapitulate ALS disease course, these models represent powerful tools for elucidating early disease mechanisms and facilitating the development of disease-modifying therapies for ALS.
